# Evidence of fatal skeletal injuries on Malapa Hominins 1 and 2

**DOI:** 10.1038/srep15120

**Published:** 2015-10-13

**Authors:** Ericka N. L’Abbé, Steven A. Symes, James T. Pokines, Luis L. Cabo, Kyra E. Stull, Sharon Kuo, David E. Raymond, Patrick S. Randolph-Quinney, Lee R. Berger

**Affiliations:** 1 Department of Anatomy, University of Pretoria, Private Bag x323, 0007, Arcadia, South Africa; 2 Department of Applied Forensic Sciences, Mercyhurst University, 501 East, 38th St, Erie, PA, 16546, USA; 3 Forensic Anthropology Program, Boston University School of Medicine, 72 East, Concord St, Boston, MA, 02118, USA; 4 Department of Anthropology, Idaho State University, 921 South, 8th Ave, Pocatello, ID, 83209, USA; 5 Department of Pathology and Anatomical Sciences, University of Missouri, One Hospital Drive, Columbia, MO, 65212, USA; 6 Department of Mechanical Engineering, California State University Los Angeles, 5151 State University Drive, Los Angeles, CA, 90032, USA; 7 School of Anatomical Sciences, University of the Witwatersrand, Parktown, 2193, South Africa; 8 Evolutionary Studies Institute, University of the Witwatersrand, Private Bag 3, Wits, 2050, South Africa; 9 Centre for Excellence in Palaeosciences, University of the Witwatersrand, Private Bag 3, Wits, 2050, South Africa

## Abstract

Malapa is one of the richest early hominin sites in Africa and the discovery site of the hominin species, *Australopithecus sediba*. The holotype and paratype (Malapa Hominin 1 and 2, or MH1 and MH2, respectively) skeletons are among the most complete in the early hominin record. Dating to approximately two million years BP, MH1 and MH2 are hypothesized to have fallen into a natural pit trap. All fractures evident on MH1 and MH2 skeletons were evaluated and separated based on wet and dry bone fracture morphology/characteristics. Most observed fractures are post-depositional, but those in the right upper limb of the adult hominin strongly indicate active resistance to an impact, while those in the juvenile hominin mandible are consistent with a blow to the face. The presence of skeletal trauma independently supports the falling hypothesis and supplies the first evidence for the manner of death of an australopith in the fossil record that is not attributed to predation or natural death.

The fossilized remains of *Au. sediba* (MH1 and MH2), discovered in 2008 at the Malapa site in the Cradle of Humankind, are hypothesized to have fallen into an open pit feature^1^,^2^, indicating a plausible proximate manner of death. Unlike many paleoanthropological specimens, the fossils from the site are exceptional in terms of preservation, anatomical positioning, and general absence of subaerial weathering and carnivore activity^3^. Therefore, any visible fractures and any notable fracture pattern on the MH1 and MH2 remains may provide direct evidence for the manner of death as opposed to later taphonomic changes.

Hominin palaeontologists rarely work with complete or associated fossils that are confidently attributed to the same individual, which hinders supportable determinations on the manner of death. Early researchers posited various theories for the death of individual Plio-Pleistocene hominin specimens, including death from inter-species violence, but these early claims were unsubstantiated as distinguishing perimortem fracture damage from post-depositional changes was impossible^4^. Interpretations of hominin cannibalism, as at Atapuerca^5^ or Stw 53 from Sterkfontein^6^, may potentially document hominins’ feeding behavior. However, individual assessments on the manner of death must rely on direct osteological evidence of fatal injuries in order to differentiate them from individuals who died from other causes and were later subjected to scavenging.

Death by predation on hominins by other species has been posited from multiple lines of evidence, including general patterns of carnivore predation upon large-bodied primates and a propensity to concentrate bones of prey in cave sites^7^,^8^,^9^,^10^,^11^,^12^,^13^,^14^. The assignment of predation as a manner of death of an individual hominin specimen relies upon evidence of feeding by a given predatory taxon and the behavior typical of that taxon being the live capture and consumption of prey, instead of scavenging individuals who are already dead. Such interpretation have been applied to the Taung site, where direct predation of the Taung child by a large eagle species is supported with damage to the cranium consistent with talon marks^15^,^16^,^17^,^18^,^19^, but preparation damage may also explain some of the defects on the cranium^20^. Similarly, crocodile feeding has been posited as a reason for the death of hominin individuals and is supported by depositional context and teeth marks on bone^21^,^22^, as seen on OH 7 (Olduvai Gorge)^23^, OH 8 and OH 35^24^. However, the reinterpretation of some of the noted defects may indicate scavenging to be a more plausible scenario^25^.

The present research proposes a specific manner of death for the MH1 and MH2 individuals through the identification of possible *in vivo* fractures that support the scenario of falling while alive.

## The Site Setting

Falls from heights into limestone formations are rarely used to explain hominin and other faunal accumulations in caves within the Cradle of Humankind. Most Plio-Pleistocene caves at the Cradle of Humankind are characterized by horizontal or near-horizontal openings and chambers as seen at Swartkrans, Sterkfontein, and Makapansgat^1^,^26^,^27^,^28^. Horizontal access permitted animals, including hominins, to enter and leave the caves easily. The current fossil record supports the claims of horizontal access as badly fragmented hominin remains are routinely recovered from these cave systems, and the taphonomic alterations are often attributed to carnivore predation and scavenging^27^.

In the last four million years, the karstic landscape of the Malmani dolomites, including Malapa, experienced major physical changes. Uplift and river incision degraded older landscape surfaces and denuded cover rocks, which exposed underlying dolomite caves^1^,^3^. Landscape modifications resulted in the creation of vertical openings, sinkholes and caverns. Open vertical shafts serve as natural traps that appear to offer shelter, water and food, but the acquisition of these essential items comes at the risk of an accidental fall^29^,^30^,^31^. Different approaches have been taken to assess faunal accumulations within natural traps, in particular, the contextual evaluation of the trap, demographic profile, and occasionally, fracture patterns of the assemblage^30^,^31^,^32^.

Based on the changing geological landscape described above, the Malapa hominins may have fallen into an opening in the limestone, possibly between 5 to 10 m in depth^1^. The potential impact surface was likely a sloping pile of gravel, sand and bat guano^1^. Therefore, both hominins would have impacted a substrate with a moderate to high energy-absorbing capacity. Rapid natural interment after deposition into the lower chamber of the cave prevented carnivore access and dispersal and, along with the pH of the deposits of the talus, may explain the hominins’ excellent preservation^1^,^2^,^26^. The location of MH1 and MH2 in their final burial location suggests that the hominins entered the cave and died within the same general time period. Following their natural burial, the roof of the lower cave chamber collapsed and the cave was subjected to repeated rock falls. Later, the cave filled up with mud and, eventually, other hominin and non-hominin material^1^.

However, fragmentary remains in the paleoanthropological record can be associated with multiple post-depositional sources of breakage, including trampling, burial, flooding, rock falls and fossilization processes – not all bone breakage is necessarily indicative of trauma associated at or near the time of death^1^,^33^,^34^,^35^,^36^,^37^. Bone biomechanics, the overall fracture patterns observed on the hominins, and the context in which the remains were discovered must be considered to create an accurate analysis of injuries^38^,^39^,^40^,^41^.

### Bone Biomechanics as a Means of Skeletal Interpretations

The combination of inorganic hydroxyapatite crystals (~65%) and organic matrix of collagen fibers (~35%) makes bone tissue a non-homogeneous, anisotropic and viscoelastic material. As a result, the response and fracture resistance of bone to externally and internally applied forces depends on the location (non-homogeneity) and direction (anisotropy) of the applied forces, as well as on the rate and velocity of loading (viscoelasticity). The flexural rigidity of bone tissue (its resistance to bending under a load) depends on all of these properties and notably leaves an imprint on the observable fracture pattern resulting from a traumatic load, mainly as a consequence of the different behavior of bone tissue under tensile and compressive forces^42^,^43^,^44^,^45^.

When fresh bone bends beyond its physiological threshold, dense cortical bone fails first into the areas experiencing tensile stress, with the fracture progressing along principal shear planes in areas of compressive stress^42^,^43^,^44^,^45^. Characteristic and diagnostic new fractures and micro-fractures appear in response to the resultant shearing, tearing and crushing of the material^38^,^39^,^42^,^43^,^44^,^45^. If the fracture surface is present and well preserved, it is possible to distinguish macroscopic characteristics of tensile and compressive failure, which in turn allows the inference of fracture dynamics and the direction of bending. These detailed analyses are possible in modern forensic cases where the bodies are well preserved, but are more difficult in archaeological or paleontological sites where the fracture surfaces have been subjected to additional taphonomic alteration for hundreds or even millions of years.

**Figure 1:**
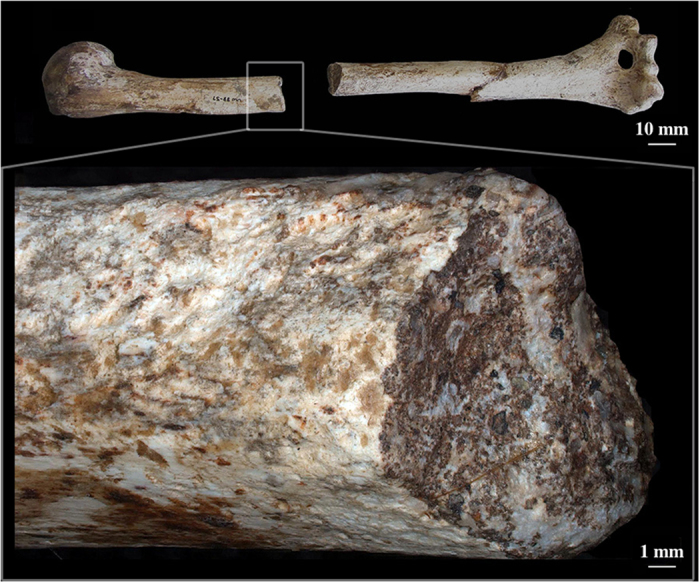
Class III (dry bone) fracture on the midshaft of the right humerus of *Au. sediba* (MH2); close-up. The fracture is not consistent with perimortem blunt force injury.

Fossil assemblages present additional difficulties for this type of interpretation, as fossil and subfossil bone is exposed to many additional sources of deformation. In fossilized bone, the original tissue has been replaced by a completely new material with radically different mechanical properties, resulting in fracture dynamics unlike those observed in either fresh or dry bone. For example, hard, completely fossilized bone can be expected to behave as a homogeneous stiff material, similar to rock, fracturing after little or no elastic deformation^38^,^39^. What once represented a variety of biological structures with differing responses to stress is now a uniform material (Fig. 1). The main reason for failure in fossilized remains is sedimentary pressure, which represents a slow compaction of bone with no added internal loads due to active musculature contractions, and no dynamic reaction of bone tissue because of fossilization.

Apart from the biomechanical properties of bone tissue, the morphology and anatomical position of a skeletal element also dictates its response to external loads *in vivo*. For example, in bipedal locomotion the upper and lower limbs provide dynamically different types of movement and respond to external stresses according to their function. When the anatomical structures are active and functional, the individual bones in each of these limbs do not react to loads and stressors in isolation but rather as a system of bones capable of transmitting loads to one another via joints and muscles. The same applies to traumatic loads. A living person has the ability to contract muscles and adapt his/her posture to oppose and mitigate an impact, which can modify the loading pattern and resulting stress distribution within and across skeletal elements. This results in distinctive fracture patterns that, when the different skeletal elements are interpreted not in isolation but as a functional system, allow one to infer and trace shared loads transmitted through joints and musculo-ligamentous attachments. This type of inference requires different skeletal elements to be confidently attributed to the same individual, which is uncommon in the fossil record.

To make an inference on the possible timing of injuries from fossilized remains, three different classes of injuries (Class I to III) were created, and ranged from macro and microscopic characteristics of fresh (*in vivo*) injuries to dry fractures in fossilized material (see Table 1 and Methods).

## Results

**Figure 2:**
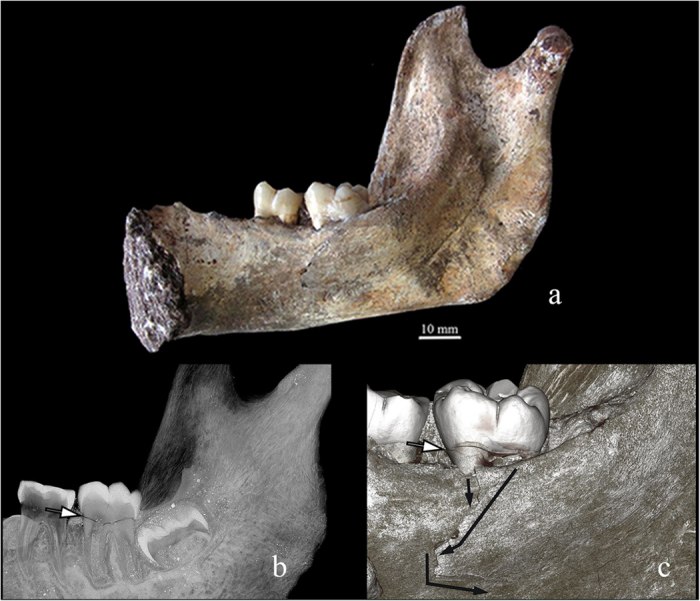
Class I fracture diagnostic of blunt force injury noted on the internal mandible and dentin of the right second lower molar of MH1: (a) overall image of the entire surface of the internal mandible, (b) computed tomography image, (c) vertical cross-section, which displays the fracture traversing through the entire crown.

**Figure 3:**
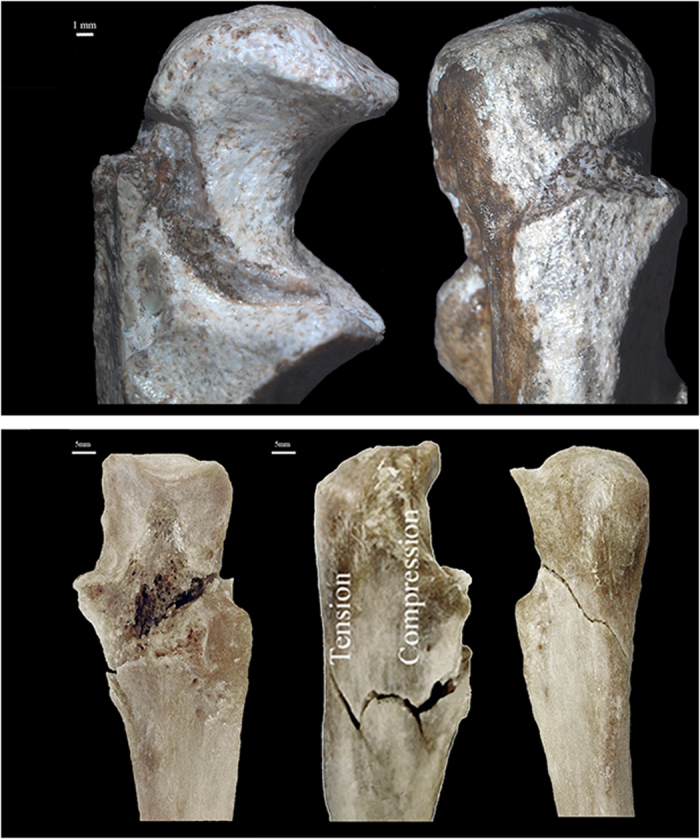
Class I fracture diagnostic of blunt force injury of the olecranon process and trochlear notch of the proximal right ulna of *Au. sediba* (MH2 top row) – two views: lateral (left) and posterior (right). While breccia is present in the Class I fracture, the modes of bone failure (tension and compression) are visible. Compare with bottom row, three views of left proximal ulna with fracture of olecranon process (anterior, lateral posterior views), which is associated with a fall from a height.

The two *Au. sediba* individuals display post-sedimentary tectonic fractures as a result of their fossilization and extended burial time (Fig. 1, Table 1). The alterations are not consistent with perimortem, fresh bone trauma (Figs 2 and 3, Table 1). Both hominins display fractures diagnostic of breaks in fresh, elastic bone – characteristic microscopic morphology was observed on the fracture surfaces and made it possible to discern between areas of tension and compression and so the direction of bending and fracture dynamics^38^,^39^.

### MH1

**Figure 4:**
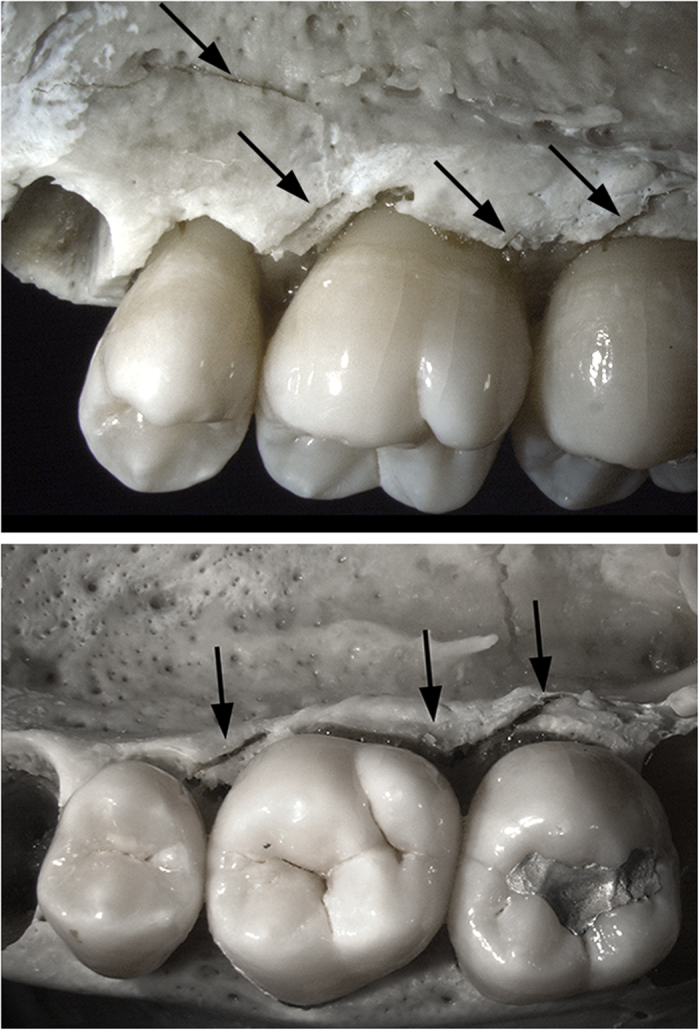
Modern forensic case and resembling alveolus failure inFig. 2. Perimortem buccal view of the upper right premolar, first and second molar alveolus bone failure (black arrows) caused by blows to the right side of the face.

**Figure 5:**
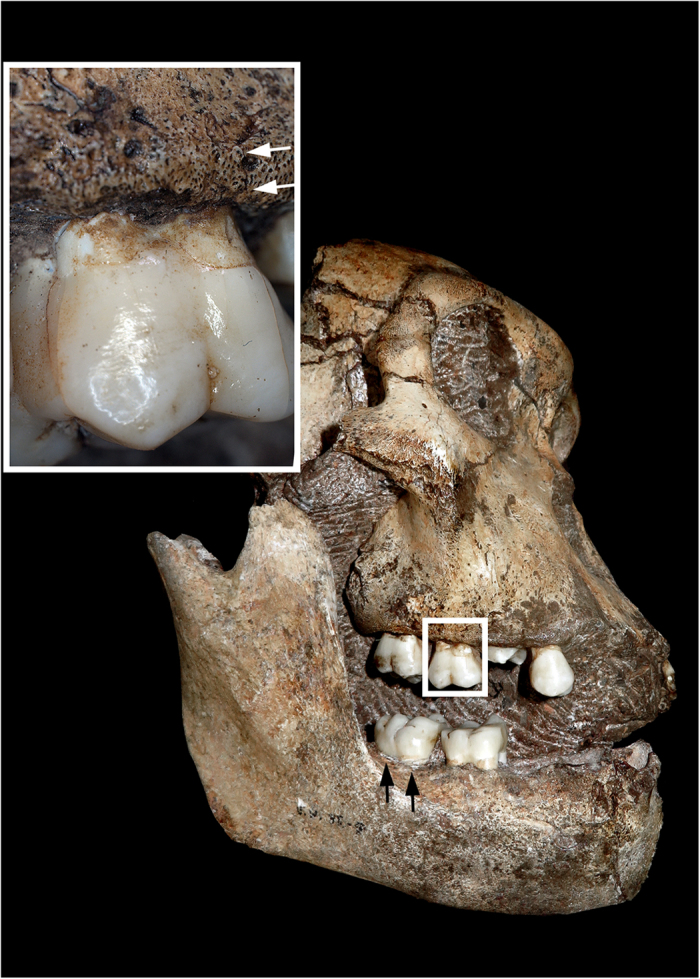
Fractures on the maxillary first molar of MH1 which are highly consistent with blunt force injuries (Class II). The upper first molar has a hairline fracture on the alveolar bone (indicated by white arrows). Crushing fractures extend through the occlusal enamel, with radiating fractures in bone adjacent to the mesial-labial root. The classification is Class II, since the biomechanics of a fracture cannot be established in enamel. The black arrows indicate the external surface of the inferior mandible where a diagnostic (Class I) fracture was noted on the internal jaw and dentin of the lower molar of MH1 (see Fig. 2).

Diagnostic biomechanical fresh bone failure (Class I) is present on the alveolus adjacent to and including both lingual roots (mesial and distal) of the right, second lower molar (Fig. 2, compare with Fig. 4). Axial as well as lateral-to-medial external loading conditions are likely responsible for fractures on the mandible and tooth (right, second lower molar [M_2_]). The most prominent area of the fracture is a 2 cm defect that traverses the thin bone of the inner mandible with an inward deformation (Fig. 2). The initial bone failure corresponds to each lingual root on M_2_ (Fig. 2b), which suggests that the roots acted as stress risers to produce the perimortem fracture. Additional damage is registered on the tooth itself, where a smooth, fresh-looking horizontal fracture essentially follows the cemento-enamel junction, separating the unsupported crown from the buttressed roots (Fig. 2b,c). Collateral damage may reflect as a highly consistent fracture (Class II) on the enamel of the corresponding right, first upper molar and a hairline fracture on its external alveolus (Fig. 5). The occlusal crown enamel fracture is considered highly consistent with perimortem injury, but biomechanical properties cannot be observed in tooth enamel and the hairline fracture of the adjoining alveolus conceals all fractured surfaces. The remaining skeletal failures on MH1 are not consistent with perimortem injury (Class III) and represent static loading and crushing as a result of transverse shifts from sedimentary pressure and other post-depositional forces (Table 1).

### MH2

The fracture patterns on MH2’s right upper limb reveal active bracing during an injury. In particular, an indirect fracture of the right olecranon process and trochlear notch of the ulna is typical of functional articulation at the time of injury, although the pattern is consistent with other scenarios. First, the contraction of the triceps muscle while undergoing forced flexion, such as in a direct blow to the elbow during a fall, may result in a bending fracture involving both the trochlear notch and olecranon process of the ulna (Fig. 3). A direct mechanism involving an impact to the olecranon could be considered, but this injury typically results in a more complex fracture pattern.

**Figure 6:**
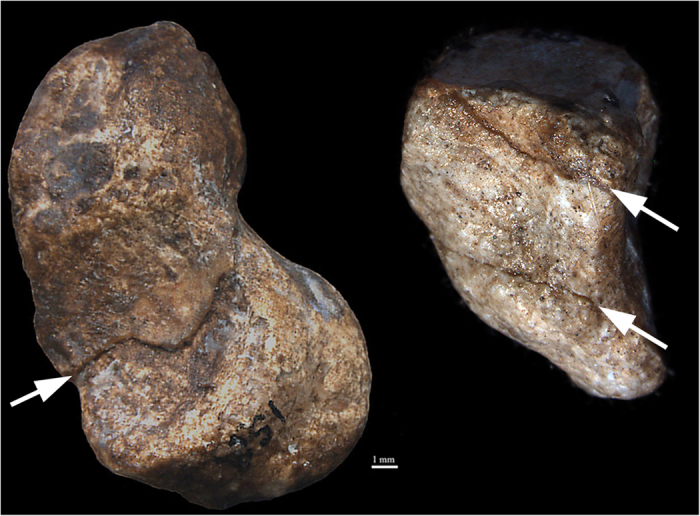
Class II fractures highly consistent with perimortem injury on right wrist (scaphoid and triquetral) *Au. sediba* (MH2) (arrows).

Fractures on the right side of the body are highly consistent with a fall from a height and include the head and neck of the first and second ribs, the head and proximal shaft of the radius, scaphoid and triquetral (Fig. 6). While no clear evidence of tensile and compressive modes of failure are noted on the ribs, the location of possible fractures on the heads of the first and second ribs suggests functional articulation to the vertebral column at the time of injury, with the head of the rib being lifted back and away from the body midline. The location and pattern of these injuries are highly consistent with an axial load to the forearm and hand, paired with an impact to the chest. This fracture pattern suggests that the hominin attempted to brace itself during a fall (Table 1).

## Discussion

The overall bone fracture morphology and patterns are highly consistent with blunt force trauma from a fall from a height, including active bracing against a fall in one individual (MH2). This patterning supports both the hypotheses of falling while alive and receiving a blunt force impact prior to death for the Malapa hominins examined.

Other possible post-depositional taphonomic causes for the above-mentioned injuries can be eliminated on contextual and morphological grounds. Weathering alteration to bone progresses as a fine network of surface cracking generally parallel to the osteon structure, with the cracks penetrating deeper as weathering proceeds to the later stages and the bone loses its structural cohesion^46^. Only traces of possible beginning weathering alteration were noted among the MH1 and MH2 remains, and none in conjunction with the observed fractures. Similarly, the fractures are inconsistent with drying cracks, which form as fresh bone dries and shrinks. Drying cracks often form in long bones parallel to the longitudinal axis and do not tend to split bones into two parts^47^. Breakage on account of carnivore gnawing was also eliminated on account of a complete lack of associated tool marks and on the location of the fractures^13^,^48^.

The excellent condition, completeness and partial articulation of the Malapa hominins made a thorough trauma analysis feasible and permitted identification of fresh bone injuries in the fossil record. Fresh and dry bone fractures can be deciphered on fossilized remains provided that the necessary preservation exists from which to view these injuries and the mechanical properties of bone are appreciated. While the number of diagnostic fractures identified on the hominins is relatively small in comparison to postmortem damage, the most parsimonious explanation for the observed trauma patterns within the discussed context is a fall from a height.

The fracture pattern in the upper limb of the adult female displays characteristics typically associated with an active response to an accidental fall, and the concurrent defects are hard to explain through other common scenarios. The location of possible fractures on the hand further suggests resistance to a fall. In modern clinical situations, the most common reason for a fractured scaphoid and triquetral is from a fall onto an outstretched hand^49^,^50^,^51^,^52^,^53^. At the time of its upper limb fracture, the female hominin (MH2) was likely to have been alive. Although the noted perimortem fractures were unlikely to have been fatal by themselves, no macroscopic signs of healing were noted – consequently, death followed in the near interval after these injuries.

While the dentition and maxilla of MH1 provide less diagnostic evidence of a fall, blunt force injuries to MH1’s face are consistent with this and other scenarios involving potential blows to the face, but the falling of objects on top of the hominin while alive or while the bone was still fresh cannot be excluded. Dental fractures, including horizontal and vertical root fractures and injury to the alveolar bone, have been documented in association with falls and blows to the face in modern forensic cases (Fig. 4. compare to Fig. 2 and Fig. 5) – horizontal root fractures are commonly noted in traumatic injuries of the teeth^54^,^55^.

Our interpretation of the death scene in this particular case does not rule out interpersonal violence as a source of perimortem trauma, but in the present depositional context, a severe injury caused by a fall from a height and followed by death is the most plausible scenario.

The greatest risk of mortality and injury for hominins was likely derived not just from predation or disease but also from accidents. The present study illustrates how the examination of fracture patterns observed on the recently deceased and the techniques employed to study and assess trauma produced *in vivo* can assist in the interpretation of relevant questions in the fossil record. Although the present study details the skeletal trauma of just two individuals, the same principles can be applied to similar sites to assess issues such as whether a hominin assemblage can be attributed to natural causes or intentional deposition. In this manner, the application of skeletal trauma analysis and bone biomechanics to fossilized remains can decisively improve our understanding of the different trauma patterns expected from different depositional modes, allowing for inferences related not only to site formation processes but also to hominin behavior.

## Methods

The first *Au. sediba* fossils were discovered in 2008 at the Malapa site in the Cradle of Humankind in South Africa^1^. Dated to approximately 1.98 million years ago^3^,^56^,^57^, the first two individuals recovered at this locality – MH1, a juvenile male, and MH2, an adult female – represent the species holotype and paratype, respectively. The two fossils were directly associated and partially articulated, representing several areas of the skeleton^1^. That each set of remains is attributed to a single individual is extremely important, as *Au. sediba* exhibited a surprising mosaic set of features, combining affinities of *Homo* with an overall australopithecine body plan but with unique hand, foot and ankle morphology^56^,^57^.

### MH1

**Figure 7:**
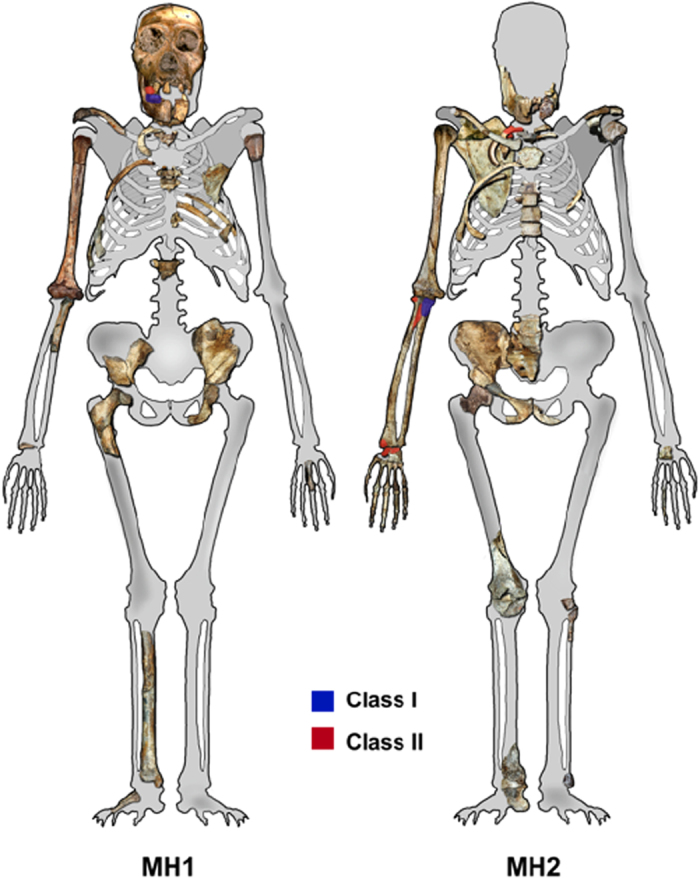
Malapa Hominins 1 and 2 inventory. Class I are diagnostic fresh bone fractures and Class II fractures are recognizable and highly consistent with blunt force injuries but unsubstantiated fractures on MH1 and MH2. Original image taken from Berger *et al.*^3^.

The skeletal remains correspond to a male juvenile estimated between 12 and 13 years old and include a partial cranium, mandible, and several fragmented and incomplete postcranial elements (clavicle, left and right humeri, eight ribs [five right, three left], a right ulna, fragments of a pelvic bone, a partial right femur, a phalanx, and three undiagnostic fragments that are probably from the lower limbs) (Fig. 7)^1^.

### MH2

The skeletal remains represent an adult female and are slightly more complete than the juvenile male. Skeletal elements present include isolated maxillary teeth, a partial and fragmented mandible, and a partial postcranial skeleton, including a sternum, two cervical vertebrae, a left hamate and capitate, many bones from the right side of the body (clavicle, scapula, five ribs [numbers one, three, five, nine and one unknown], humerus, radius, ulna, an articulated wrist and hand, ilium, femoral head, pubic bone, and an articulated ankle [tibia, calcaneus, and talus]), and fragmentary thoracic vertebrae, sacrum, left pedal phalanges, and left tibia and fibula (Fig. 7)^1^.

Fractures were classified into three different classes (Class I to III) according to their macroscopic and microscopic characteristics of fresh or dry bone, as well as their anatomical location (Table 1). Fracture classifications were in line with the standard procedures of the “Istanbul Protocol Manual on the Effective Investigation and Documentation of Torture and other Cruel, Inhuman or Degrading Treatment or Punishment”^58^. Class I fractures were considered diagnostic of blunt force injury and “could not have been caused in any way other than that described”^58^; Class II fractures are “highly consistent with blunt force injury but few other causes for the fracture” are possible^58^; and Class III fractures are “not consistent with perimortem blunt force injuries”^58^. Fractures were evaluated and photographed with a Zeiss Axio Zoom v.16 stereoscope. Potential fractures on MH1 and MH2 were compared to fracture patterns on two modern forensic cases, including a fall from heights down a steep ravine and a blow to the face.

Class I fractures are diagnostic of a dynamic loading (low velocity impact) on green, elastic bone, including clearly recognizable and delimited regions of failure under either tension or compression, allowing the inference of direction of bending and fracture dynamics (Table 1, Figs. 2 and 3). The loss of bone collagen (elasticity) and time span for fractures occurrence varies tremendously and is based on the depositional context of the remains^59^,^60^. Class I fractures would provide the most unequivocal evidence regarding potential deposition scenarios, especially when green-bone reaction is combined with fracture location and dynamics (Table 1, Fig. 7). Class II fractures display macroscopic outlines and anatomical locations highly consistent with a typical dynamic load *in vivo* (Table 1, Fig. 7), but whose edges and fracture surfaces are altered or simply not visible for assessment, not allowing inferences on the exact fracture dynamics, as tension and compression regions are ambiguous (Table 1). In spite of this limitation, Class II fractures allow the consideration of possible injury patterns even if post-depositional processes have erased or compounded diagnostic markers of the precise mode of failure (Table 1, Fig. 5 and 6). In particular, rather than considered in isolation, Class II fractures are significant when they suggest consistent load patterns across different, articulating skeletal elements. Brittle, likely post-depositional fractures (hence not relevant to infer the manner of death) are grouped in Class III (Table 1, Fig. 1).

## Additional Information

**How to cite this article**: L’Abbé, E. N. *et al.* Evidence of fatal skeletal injuries on Malapa Hominins 1 and 2. *Sci. Rep.* **5**, 15120; doi: 10.1038/srep15120 (2015).

## Figures and Tables

**Table 1:** Three fracture categories applied to MH1 and MH2 (Fig. 7).

Fracture Class	Definition	Characteristics
I	Diagnostic fractures of blunt force injury. Fresh (organic) bone, with well preserved, clear microscopic morphology.	Low velocity impact, plastic deformation – consistent with a dynamic load (Figs 2 and 3).
II	Highly consistent fractures of blunt force injury. Outline and location are typical of *in vivo* breakage, but fracture mode and dynamics cannot be conclusively substantiated.	Tension/compression markers cannot be identified, but the fracture morphology and anatomical pattern are highly consistent with those observed in common, modern-day injuries (Fig. 4, compare Fig. 5). Also see Fig. 6.
III	Dry, static bone fractures not consistent with perimortem blunt force injury.	Sedimentary pressures and other post-depositional changes (Fig. 1).
